# Impact of nonsurgical periodontal treatment on arterial stiffness outcomes related to endothelial dysfunction: A systematic review and meta‐analysis

**DOI:** 10.1002/JPER.24-0422

**Published:** 2024-11-16

**Authors:** Alessandro Polizzi, Luigi Nibali, Gianluca Martino Tartaglia, Gaetano Isola

**Affiliations:** ^1^ Department of General Surgery and Medical‐Surgical Specialities Unit of Periodontology, University of Catania Catania Italy; ^2^ Periodontology Unit, Centre for Host Microbiome Interactions, Faculty of Dentistry, Oral & Craniofacial Sciences King's College London London UK; ^3^ Fondazione IRCCS Cà Granda Ospedale Maggiore Policlinico Milan Italy; ^4^ Department of Biomedical, Surgical, and Dental Sciences University of Milan Milan Italy

**Keywords:** arterial stiffness, carotid intima‐media thickness, flow‐mediated dilatation, meta‐analysis, nonsurgical periodontal treatment, periodontitis, pulse wave velocity

## Abstract

**Background:**

To assess the available evidence on whether nonsurgical periodontal treatment (NSPT) improves arterial stiffness outcomes in patients with periodontitis (PD).

**Methods:**

Following the Preferred Reporting Items for Systematic Reviews and Meta‐analyses (PRISMA) guidelines and population, intervention, comparison, outcomes, and study design (PICOS) question, electronic databases were screened for clinical interventional studies addressing the impact of NSPT on pulse wave velocity (PWV), carotid intima‐media thickness (CIMT), and flow‐mediated dilatation (FMD) outcomes in PD patients. Furthermore, the research strategy was implemented using a hand search. Studies were selected, and data were extracted by two independent reviewers. Random effects models were applied to perform a meta‐analysis, and methodological index for nonrandomized studies (MINORS) and Cochrane Rob2 tools were used to assess the risk of bias.

**Results:**

Fifteen articles were finally included for qualitative synthesis. Among them, eight single‐arm cohort studies met the final inclusion criteria for meta‐analysis. The Rob2 analysis evidenced that one randomized clinical trial (RCT) had a low risk, three RCTs raised some concerns, and three RCTs had a high risk of bias, while the MINORS scores ranged from 9 to 14. The meta‐analysis showed that NSPT significantly impacted FMD (*p* < 0.001) and CIMT (*p* = 0.004), while changes in PWV were not statistically significant. However, there was high heterogeneity among studies (*I* ^2^ = 78% for FMD and *I* ^2^ = 62% for CIMT).

**Conclusion:**

Despite some beneficial effects on FMD and CIMT, due to study limitations, high heterogeneity, and risk of bias, it cannot be concluded that NSPT is effective in improving arterial stiffness. Therefore, further studies are necessary to achieve high‐quality evidence on the effect of NSPT on arterial stiffness outcomes in PD patients.

**Trial registration:**

PROSPERO ID CRD42024501399.

**Plain Language Summary:**

Periodontitis (PD) has been associated with alterations in arterial stiffness outcomes related to early endothelial dysfunction. Based on noninterventional studies, this meta‐analysis indicates that nonsurgical periodontal treatment (NSPT) may reduce cardiovascular disease risk in patients with PD. The moderate evidence derived from the studies that were finally included showed that NSPT had beneficial effects on flow‐mediated dilatation and carotid intima‐media thickness, while this trend was not observed for pulse wave velocity. Moreover, the findings of the present meta‐analysis were characterized by high heterogeneity and risk of bias and were derived from uncontrolled clinical trials or randomized clinical trials with limitations. Therefore, more studies with standardized protocols and homogeneous arterial stiffness outcomes are needed to elevate the quality of the present evidence.

## 1 INTRODUCTION

Periodontitis (PD) is a chronic inflammatory disease characterized by a dysregulated host inflammatory response against dysbiotic oral biofilm that, if not properly treated, can cause progressive destruction of the tooth‐supporting tissues, leading to tooth loss.^1^

Mounting evidence suggests that PD, with its chronic burden on periodontal tissues, may serve as a potential negative stimulus to cardiovascular diseases (CVDs).^2^, ^3^, ^4^ In this regard, endothelial dysfunction, a phenomenon characterized by impaired endothelium‐dependent vasodilation and a proinflammatory/prothrombotic state, has been shown to have a central role in the pathogenesis and evolution of both atherosclerosis and CVD.^5^, ^6^ For endothelial dysfunction, arterial stiffness dysregulation also represents an important feature in the development of CVDs through its role in the reduced elasticity of arteries, which impairs their ability to expand and contract in response to blood flow,^7^ together being an indicator of cardiovascular status in apparently healthy patients.^8^ Several studies indicated some effects on direct and indirect outcome measures of arterial stiffness, including flow‐mediated dilatation (FMD), carotid intima‐media thickness (CIMT), and pulse wave velocity (PWV), all of them potentially associated with an increased risk of CVD in patients with PD.^9^, ^10^

In this context, nonsurgical periodontal treatment (NSPT), using different hand and ultrasonic instrumentations methods,^11^, ^12^, ^13^ has been recently reported as one of the first approaches for the treatment of PD patients^14^; however, while the effects of NSPT approaches^14^, ^15^ on periodontal health are well‐documented, its related systemic effects, particularly on endothelial function,^2^ remain a subject of ongoing investigation and debate. Studies on surrogate indicators for cardiovascular outcomes indicated that NSPT was able to improve FMD^16^; conversely, CIMT was found to be reduced in a preliminary interventional study,^17^ while it was not improved following NSPT in a multicentric study.^18^ Similarly, conflicting results were reported on the impact of NSPT on PWV 6 months after NSPT.^19^, ^20^

In light of the abovementioned evidence, the present systematic review and meta‐analysis were aimed at evaluating the current evidence on the efficacy of NSPT on arterial stiffness outcomes related to the risk of early endothelial dysfunction in PD patients.

## 2 MATERIALS AND METHODS

### 2.1 PICOS question, inclusion criteria, and research strategy

This systematic review and meta‐analysis followed the Preferred Reporting Items for Systematic Reviews and Meta‐analyses (PRISMA) guidelines^21^ (see Table S1 in the online *Journal of Periodontology*) and was registered in the PROSPERO database under registration number CRD42024501399. The population, intervention, comparison, outcomes, and study design (PICOS) components were the following:

1. Population: adult patients diagnosed with PD (excluding individuals with PD as a manifestation of systemic disease or necrotizing PD) who have undergone NSPT.
2. Intervention: NSPT intended as subgingival scaling and root planing or root surface debridement.
3. Comparison: supragingival ultrasonic debridement or no treatment, where applicable. In the case of the use of adjunctive treatments, NSPT without antibiotics or with placebo was considered as control.
4. Outcome measures: endothelial dysfunction and arterial stiffness evaluated in terms of PWV, CIMT, and FMD:
  - PWV, defined as the speed of pulse wave propagation between two known sites, commonly carotid‐femoral (CfPWV) or carotid‐radial (CrPWV) pulses. It is measured with pressure sensors, such as the tonometer,^9^ and is obtained by the ratio of the distance between the two recording sites in a vascular segment traversed by pressure waves divided by the time required for the pressure wave to travel this distance (PWV = distance/transit time).^22^
  - CIMT, defined as the distance between two lines visible from carotid B‐mode ultrasound: an upper one indicating the interface between the lumen and intima and a lower one identifying the interface between the medial layer and the adventitia layer. CIMT is usually measured in the vicinity of the carotid bifurcation in the internal carotid artery.^22^
  - Endothelium‐dependent FMD, defined as the vasodilator response of a conduit artery to elevations in blood flow‐associated shear stress after 5 min of ischemia, assessed by high‐resolution ultrasound.^23^
5. Study design: randomized and nonrandomized controlled trials, cohort studies, case–control studies, and retrospective studies. Intervention studies in the English language assessing the impact of NSPT on indicators of arterial stiffness (PWV, FMD, CIMT) in subjects with PD have been included.

The search strategy for the detection of all relevant publications on this topic was conducted by combining Medical Subject Heading (MeSH) terms and free text words with Boolean operators (“AND,” “OR”) on the following databases: PubMed, Scopus, Web of Science, and LILACS (see Table S2 in the online *Journal of Periodontology*).

### 2.2 Exclusion criteria

Observational studies without interventional protocol and not related to the impact of NSPT on the primary outcomes of the present meta‐analysis (PWV, FMD, and CIMT) were excluded. Furthermore, the following were not included either: case reports/series, opinion articles, theses, narratives, and systematic reviews.

### 2.3 Selection process and data extraction

Records identified through database searches underwent an initial screening by two independent reviewers (A.P., G.I.); potentially relevant articles were selected based on information from titles and abstracts. The articles evaluated as eligible by both reviewers underwent a further review in the full‐text versions for adherence to the inclusion criteria. Cohen's kappa statistic was calculated to assess interobserver agreement for the article selection process. A hand search (without a timeline setting) was complemented in the following journals: *European Heart Journal, Journal of Dental Research, Journal of Clinical Periodontology, Journal of Periodontology, Journal of Periodontal Research*, and *Periodontology 2000* from January 1, 2004 to May 2, 2024.

The data extracted from the studies included in the review were: authors, year of publication, aim of the study, design of the study, sample, NSPT protocol, main outcomes, baseline values, values after periodontal treatment, and conclusions. In studies with a mixed design (observational case–control + interventional for cases), the sample size and study design were considered, taking into account the interventional part that solely corresponded to the study's present objectives. Disagreements between the review authors over the selection of specific papers were resolved by discussion, with the involvement of a third review author (G.M.T.) where necessary.

### 2.4 Risk of bias

Two reviewers (A.P., G.I.) independently assessed the risk of bias in included studies using the methodological index for nonrandomized studies (MINORS) tool for single‐arm clinical trials^24^ and the Cochrane Rob2 tool for randomized clinical trial (RCT) studies.^25^ Disagreements between the review authors over the risk of bias in particular studies were resolved by discussion, with the involvement of a third review author (G.M.T.) where necessary.

### 2.5 Meta‐analysis

A narrative data synthesis was carried out first. Meta‐analyses were attempted in the presence of at least two papers with similar methodology and reporting the same outcomes. Heterogeneity was assessed using the *χ* ^2^‐based Q‐statistic method, considered significant if *p* < 0.05, and quantified with the *I* ^2^ statistic. The pooled estimates of the mean differences (MDs) in the main outcomes were calculated using random effects models to consider potential interstudy heterogeneity and adopt a more conservative approach. The pooled effect was considered significant with a *p* value <0.05. Forest plots with 95% confidence interval (CI) and heterogeneity (Cochrane's *Q* test and the *I* ^2^ statistic) were calculated using statistical software.*

## 3 RESULTS

### 3.1 Identification of eligible articles

**FIGURE 1:**
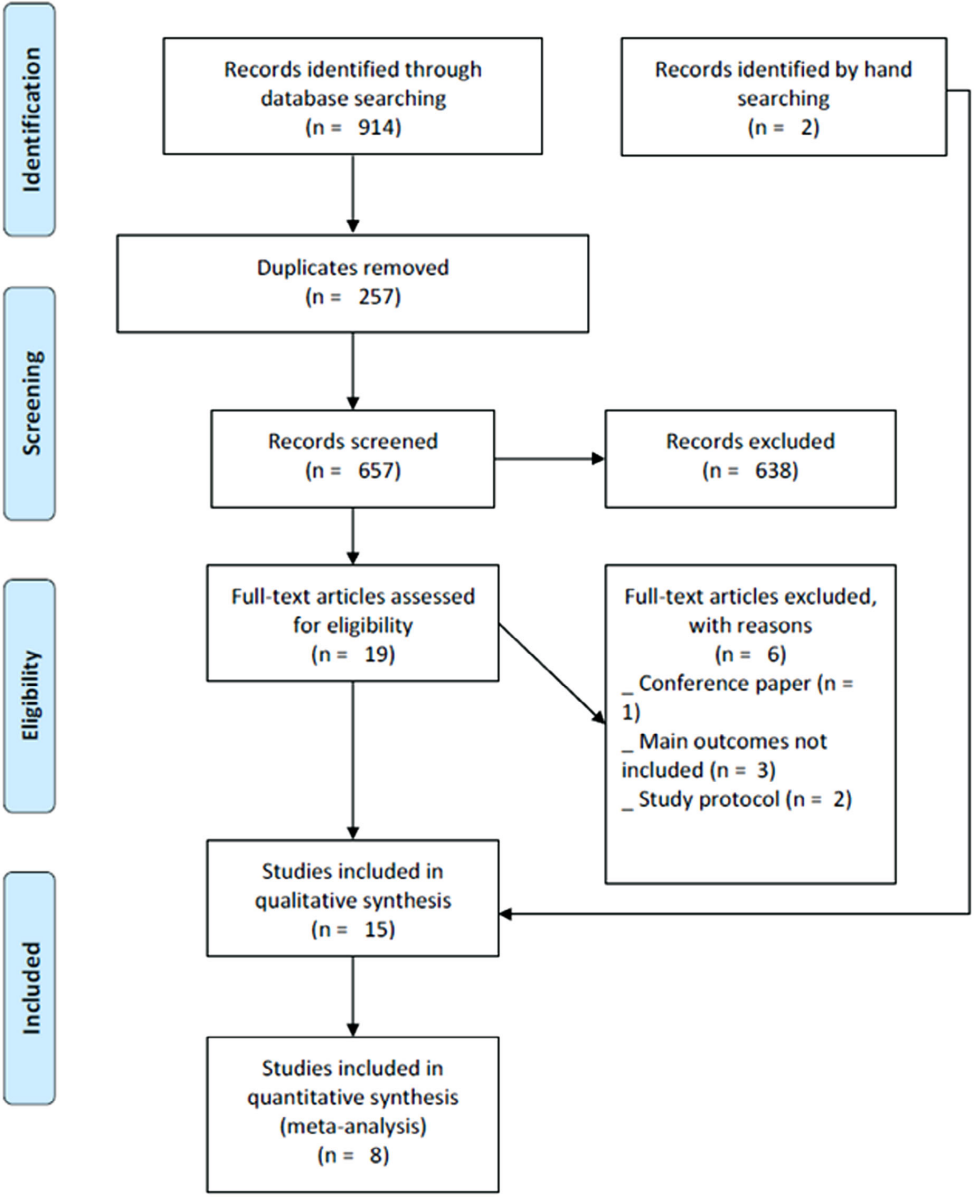
PRISMA flow diagram of the study. PRISMA, Preferred Reporting Items for Systematic Reviews and Meta‐analyses.

The database search initially identified a total of 914 records: 178 from PubMed, 355 from Scopus, 359 from Web of Science, and 22 from LILACS (see Table S2 in the online *Journal of Periodontology*). After removing 257 duplicates, 657 records remained for title and abstract screening. Of these, 638 papers were excluded, and 19 articles were assessed for full‐text eligibility. Six studies were excluded for the following reasons: being a conference paper (*n* = 1), main outcomes not included (*n* = 3), and study protocol (*n* = 2) (see Table S3 in the online *Journal of Periodontology*). Ultimately, 15 articles^12^, ^17^, ^18^, ^19^, ^20^, ^26^, ^27^, ^28^, ^29^, ^30^, ^31^, ^32^, ^33^, ^34^, ^35^ were included for qualitative synthesis and 8 were suitable for meta‐analysis^17^, ^18^, ^19^, ^26^, ^30^, ^33^, ^34^, ^35^ (Figure 1). Cohen's kappa statistic results showed good interobserver reliability for both the abstract screening (*k* = 0.88) and the full‐text analysis stages (*k* = 0.94).

### 3.2 Qualitative synthesis of studies

Table 1 summarizes the data extracted from the included studies. Eight articles^17^, ^18^, ^19^, ^26^, ^30^, ^33^, ^34^, ^35^ were single‐arm cohort studies, whereas the remaining seven were randomized controlled clinical trials (RCTs).^12^, ^20^, ^27^, ^28^, ^29^, ^31^, ^32^ In the included RCTs, the control groups were represented by supragingival scaling/debridement,^12^, ^27^, ^31^ no treatment,^29^, ^32^ NSPT without antibiotics,^28^ or NSPT + placebo.^20^ The number of patients included in these prospective studies ranged from 13 to 290.^26^, ^31^ Ten studies included patients with both mild and severe PD without comorbidities. However, some authors included participants with comorbidities such as hypertension,^19^, ^27^ diabetes,^31^ stable coronary disease,^32^ and rheumatoid arthritis.^33^ Moreover, in some studies, adjuvant treatments were used, including systemic antibiotics^20^, ^26^, ^28^ and local antiseptics.^27^, ^31^ There was a high heterogeneity in the follow‐up periods after NSPT, which ranged from 2 months^27^ to 3 years.^18^

**TABLE 1:** Characteristics of included studies.

Study	Aim	Design of study	Sample	Comorbidities	Treatment	Baseline outcomes	Follow‐up	Main findings
*Czesnikiewicz‐Guzik M, et al. 2019*	To conduct RCT to investigate how periodontal disease therapy affects endothelial function	RCT	101 patients with PD	Hypertension	(1) NSPT + chlorhexidine 0.2% gel vs. (2) supragingival scaling	(1) FMD: 7.6% (2) FMD: 7.2%	(1) FMD (2 months): 10% (2) FMD (2 months): 7.9%	Participants in IPT showed higher FMD 2 months after therapy than those receiving standard supragingival debridement
*Houcken W, et al. 2016*	To investigate how periodontal treatment affects stiffness of arteries in PD patients	RCT	45 patients with PD (20 treated with adjuvant systematic antibiotics)	None	(1) NSPT (2) NSPT + amoxicillin 375 t.i.d. and metronidazole 500 mg t.i.d. for 7 days	PWV^a^: 8.00 ± 1.8 m/s	PWV (6 months)^a^: 7.82 ± 1.6 m/s	Despite lack of substantial reduction in arterial stiffness following periodontal therapy, a little decrease in systolic blood pressure was noted after 6 months. Results of subanalysis on patients who received systemic antibiotics as adjuvant did not vary from overall findings
*Jockel‐Schneider Y, et al. 2017*	To evaluate how anti‐infective periodontal treatment affects state of vascular health	RCT	55 patients with SPD	None	(1) NSPT + amoxicillin 500 mg and metronidazole 400 mg t.i.d. for 7 days (2) NSPT + placebo	PWV^a^: 8.92 ± 2.23 m/s	PWV (12 months)^a^: 8.85 ± 2.27 m/s	Overall PWV values at baseline and after 1 year were comparable. After 1 year, patients were evenly divided into three groups according to tertiles of BoP resolution, and best response tertile (ΔBoP ≥ 88%) showed significant drop in observed PWV median value of −0.6 m/s (*p* < 0.04)
*Kapellas K, et al. 2014*	To evaluate if NSPT could enhance measurements of artery function	RCT	168 patients with PD	None	(1) Full‐mouth NSPT (2) No treatment	CIMT (1): 0.64 ± 0.14 mm PWV (1): 8.27 ± 1.30 m/s CIMT (2): 0.64 ± 0.12 PWV (2): 8.37 ± 1.36 m/s	After 12 months: CIMT (1): 0.63 ± 0.14 mm PWV (1): 8.44 ± 0.92 m/s CIMT (2): 0.65 ± 0.11 mm PWV (2): 8.33 ± 1.04 m/s	In aboriginal Australians with PD, periodontal treatment decreased subclinical arterial thickness but did not affect function, indicating strong correlation between PD and atherosclerosis
*Rapone B, et al. 2022*	To evaluate effects of IPT on diabetes patients' lipid profiles and endothelial function	RCT	290 patients with PD	Diabetes	(1) Full‐mouth NSPT + chlorhexidine (2) supragingival debridement	FMD (1): 8.12 ± 1.00% FMD (2): 7.50 ± 0.25%	After 6 months: FMD (1): 8.23 ± 0.46% FMD (2): 8.56%^b^	Rigorous periodontal regimen may enhance endothelial function, implying a positive impact on vasculature that may be mediated by decrease in systemic inflammation. However, there were no discernible statistically significant differences between groups
*Saffi MAL, et al. 2018*	To evaluate how periodontal therapy affects coronary artery disease patients' endothelial function	RCT	69 patients with SPD	Stable coronary disease	(1) NSPT (2) No treatment	FMD (1): 7.05 ± 5.6% FMD (2): 7.10 ± 6.09%	After 3 months: FMD (1): ↑ 1.39% FMD (2): ↑ 1.37%	In brief follow‐up time, periodontal therapy did not improve vasodilation in individuals with coronary disease
*Tonetti MS, et al. 2007*	To assess over 6 months how periodontal treatment affects endothelial function	RCT	120 patients with SPD	None	(1) Full‐mouth NSPT (2) Supragingival debridement	FMD (1): 7.1 ± 4.2% FMD (2): 6.5 ± 2.6%	After 6 months: FMD (1)–FMD (2) ↑ 1.48%	Endothelial dysfunction and acute, transient systemic inflammation were outcomes of intensive periodontal therapy. However 6 months following treatment, improvements in oral health were linked to enhanced endothelial function
*Blum A, et al. 2007*	To assess if improving endothelial function and preventing cardiovascular events may be achieved by appropriate therapy of PD	Single‐arm cohort study	13 patients with SPD	None	NSPT + antibiotics (amoxicillin 500 mg + metronidazole 250 mg t.i.d.) during first week	FMD: 4.12 ± 3.96%	FMD (3 months): 11.12 ± 7.22%	In addition to improving endothelial function, treating PD can be a key CVD prevention strategy
*Kudo C, et al. 2018*	To conduct prospective, multicenter, observational research to assess how periodontal therapy affects atherosclerosis	Multicenter single‐arm cohort study	92 patients with PD	None	NSPT + SPT	PWV (8 subjects): 15.96 ± 2.69 m/s CIMT (8 subjects): 0.94 ± 0.14 mm	After 3 years (8 subjects): PWV: 17.17 ± 2.91 m/s CITM: 0.94 ± 0.14 mm	Following NSPT therapy, there was substantial decrease in both left and right max. CIMT levels at several follow‐up sessions, which was correlated with improvements in periodontal clinical indicators
*Mercanoglu F, et al. 2004*	To assess whether individuals with chronic PD have endothelial dysfunction and, if so, if treatment might help them recover	Single‐arm cohort study	28 patients with PD	None	Full‐mouth NSPT	FMD: 8.4 ± 4.0%	FMD (after 6 weeks): 17.7 ± 5.7%	Patients with chronic PD have decreased endothelial functions, which improve with starting periodontal treatment
*Piconi S, et al. 2009*	To evaluate if periodontal therapy alone might beneficially alter intima‐media thickness	Single‐arm cohort study	35 patients with PD	None	NSPT	CIMT: 0.55 ± 0.03 mm	CIMT (after 12 months): 0.45 ± 0.04 mm	Endothelial dysfunction improves and CIMT decreases after periodontal disease treatment
*Salah S, et al. 2023*	To determine whether treating PD can reduce incidence of CVD	Single‐arm cohort study	30 patients with PD	Rheumatoid arthritis	Full‐mouth NSPT	CIMT: 1.1 ± 0.2 mm	CIMT (after 6 months): 0.96 ± 0.2 mm	Patients with rheumatoid arthritis may have better cardiovascular health if PD is treated
*Seinost G, et al. 2005*	To assess if endothelial dysfunction would be improved by periodontal therapy	Single‐arm cohort study	30 patients with SPD	None	NSPT	FMD: 6.1 ± 4.4%	FMD (after 3 months): 9.8 ± 5.7%	When severe PD is treated, endothelial dysfunction is corrected. More research is necessary to determine if better endothelial function will have a positive impact on atherogenesis and cardiovascular events
*Toregeani JF, et al. 2014*	To assess how periodontal therapy affects CIMT	Single‐arm cohort study	21 patients with SPD	None	NSPT	CIMT: 0.60 ± 0.11 mm	CIMT (after 12 months): 0.58 ± 0.11 mm	After 6 months, NSPT was successful in lowering CIMT. However, following a year, CIMT levels reverted to values that were similar to the initial ones
*Vidal F, et al. 2013*	To assess how NSPT affects vascular stiffness	Single‐arm cohort study	26 patients with PD	Refractory hypertension	NSPT	PWV: 13.7 ± 2.4 m/s	PWV (after 6 months): 12.5 ± 1.9 m/s	Patients with resistant hypertension saw considerable reduction with periodontal treatment

Abbreviations: ↑, increasing; BoP, bleeding on probing; CIMT, carotid intima‐media thickness; CVD, cardiovascular disease; FMD, flow‐mediated dilatation; IPT, intensive periodontal therapy; NSPT, nonsurgical periodontal treatment; PD, periodontitis; PWV, pulse wave velocity; RCT, randomized clinical trial; SPD, severe periodontitis; SPT, supportive periodontal treatment; t.i.d., three times a day (*ter in die*).

^a^ Subgroup analysis not available.

^b^ Standard deviation not provided.

The results of FMD values in the different follow‐up sessions are presented in Table 2: four studies were RCTs,^12^, ^27^, ^31^, ^32^ whereas the remaining three were single‐arm cohort studies.^26^, ^30^, ^34^ All the RCTs evaluating FMD reported differentiated results between control and intervention. Five studies reported a statistically significant improvement in FMD after NSPT or supragingival debridement at the last follow‐up sessions. Saffi et al.^32^ reported a nonsignificant improvement in FMD in both the NSPT and control groups (no treatment) after 3 months of follow‐up, while Tonetti et al.^12^ reported a statistically significant difference between the two protocols (full‐mouth NSPT vs. supragingival debridement) after 1‐day, 2‐month, and 6‐month follow‐up sessions.

**TABLE 2:** Comprehensive summary of results of impact of periodontal treatment on FMD at different follow‐up sessions.

				FMD at follow‐up sessions							
Paper	Study design	Number of patients	Type of intervention	Baseline	1 day	1 week	1 month	6 weeks	2 months	3 months	6 months
*Czesnikiewicz‐Guzik M, et al. 2019*	RCT	101 PD with hypertension	(1) NSPT + chlorhexidine 0.2% gel vs. (2) supragingival scaling	(1) 7.6% (2) 7.2%	–	–	–	–	(1) 10%^a^ (2) 7.9%	–	–
*Rapone B, et al. 2022*	RCT	290 PD with diabetes	(1) Full‐mouth NSPT + chlorhexidine (144 PD) (2) supragingival debridement (143 PD)	(1) 8.12 ± 1.00% (2) 7.50 ± 0.25%	–	–	–	–	–	(1) 8.35 ± 0.12% (2) 8.32 ± 0.46%	(1) 8.23 ± 0.46% (2) 8.56%^b^
*Saffi MAL, et al. 2018*	RCT	69 SPD with stable CVD	(1) NSPT (2) No treatment	(1) 7.05 ± 5.6% (2) 7.10 ± 6.09%	–	–	–	–	–	(1) ↑ 1.39% (2) ↑ 1.37%	–
*Tonetti MS, et al. 2007*	RCT	120 SPD	(1) Full‐mouth NSPT (2) Supragingival debridement	(1) 7.1 ± 4.2% (2) 6.5 ± 2.6%	−1.74%^c^ ^,^ ^d^	−0.43%^d^	−0.25%^d^	–	0.86%^c^ ^,^ ^d^	–	1.48%^c^ ^,^ ^d^
*Blum A, et al. 2007*	Single‐arm cohort study	13 SPD	NSPT + antibiotics (amoxicillin 500 mg + metronidazole 250 mg t.i.d.) during first week	4.12 ± 3.96%	–	–	–	–	–	11.12 ± 7.22%^a^	–
*Mercanoglu F, et al. 2004*	Single‐arm cohort study	28 PD	Full‐mouth SRP	8.4 ± 4.0%	–	–	–	17.7 ± 5.7%^a^	–	–	–
*Seinost G, et al. 2005*	Single‐arm cohort study	30 SPD	NSPT	6.1 ± 4.4%	–	–	–	–	–	9.8 ± 5.7%^a^	–

Abbreviations: ↑, increment; ↓, decrement; CVD, cardiovascular disease; FMD, flow‐mediated dilatation; NSPT, nonsurgical periodontal treatment; PD, periodontitis; RCT, randomized clinical trial; SPD, severe periodontitis; SRP, scaling and root planing; t.i.d., three times a day (*ter in die*).

^a^ Statistically significant difference compared to baseline.

^b^ Standard deviation not provided.

^c^ Statistically significant difference between two protocols within baseline/follow‐up session.

^d^ These values are intended as difference between control and test group mean FMD.

Five articles evaluated the impact of NSPT on PWV (Table 3); three studies were RCTs,^20^, ^28^, ^29^ and two studies were single‐arm cohort studies.^18^, ^19^ PWV results were differentiated between intervention and control only in the study of Kapellas et al.^29^ Vidal et al.^19^ was the only one that found a statistically significant impact of NSPT on PWV at 6‐month follow‐up (baseline PWV 13.7 ± 2.4 m/s vs. 6‐month PWV 12.5 ± 1.9 m/s). All the results from the other articles were statistically not significant.

**TABLE 3:** Comprehensive summary of results of impact of periodontal treatment on PWV at different follow‐up sessions.

				PWV at the follow‐up sessions					
Paper	Study design	Number patients	Type of intervention	Baseline	1 day	3 months	6 months	1 year	3 years
*Houcken W, et al. 2016*	RCT	45 PD	(1) NSPT (2) NSPT + amoxicillin 375 t.i.d. and metronidazole 500 mg t.i.d. for 7 days	8.00 ± 1.8 m/s^a^	–	8.14 ± 1.9 m/s^a^	7.82 ± 1.6 m/s^a^	–	–
*Jockel‐Schneider Y, et al. 2017*	RCT	55 SPD	(1) NSPT + amoxicillin 500 mg and metronidazole 400 mg t.i.d. for 7 days (2) NSPT + placebo	8.92 ± 2.23 m/s^a^	–	–	–	8.85 ± 2.27 m/s^a^	–
*Kapellas K, et al. 2014*	RCT	135 PD	(1) Full‐mouth NSPT (2) No treatment	(1) 8.27 ± 1.30 m/s (138 PR) (2): 8.37 ± 1.36 m/s (135 PR)	–	(1) 8.15 ± 1.09 m/s (87 PR) (2) 8.18 ± 1.17 m/s (82 PR)	–	(1) 8.44 ± 0.92 m/s (89 PR) (2) 8.33 ± 1.04 m/s (79 PR)	–
*Kudo C, et al. 2018*	Multicenter single‐arm cohort study	51 PD	NSPT + SPT	15.92 ± 3.41 m/s (51 PR)	16.73 ± 3.29 m/s (25 PR)	–	–	17.30 ± 3.62 m/s (17 PR)	17.16 ± 2.91 m/s (8 PR)
*Vidal F, et al. 2013*	Single‐arm cohort study	26 PD	NSPT	13.7 ± 2.4 m/s	–	13.4 ± 2.4 m/s	12.5 ± 1.9 m/s^b^	–	–

Abbreviations: NSPT, nonsurgical periodontal treatment; PD, periodontitis; PWV, pulse wave velocity; RCT, randomized clinical trial; SPD, severe periodontitis; SPT, supportive periodontal treatment; t.i.d., three times a day (*ter in die*).

^a^ Subgroup analysis not available.

^b^ Statistically significant difference compared to baseline.

Table 4 resumes the results of the effect of periodontal treatment on CIMT at different follow‐up sessions. Four studies were single‐arm cohort studies^17^, ^18^, ^33^, ^35^ and one article was an RCT.^29^ The RCT^29^ presented a statistically significant difference between CIMT at baseline and after 1 year, without distinguishing the intervention from the control (no treatment). Regarding the cohort studies, three studies reported a significant positive impact of NSPT in CIMT after 6 months^17^, ^33^, ^35^ and 1 year.^17^ However, these differences were limited to a maximum absolute difference of 0.15 mm,^34^ 0.14 mm,^37^ or 0.06 mm^35^ at 6‐month follow‐up when compared to baseline values, while Kudo et al.^18^ did not detect any improvement in CIMT after NSPT or during supportive periodontal treatment (SPT).

**TABLE 4:** Comprehensive summary of results of impact of periodontal treatment on CIMT at different follow‐up sessions.

				CIMT at follow‐up sessions					
Paper	Study design	Number of patients	Type of intervention	Baseline	1 day	3 months	6 months	1 year	3 years
*Kapellas K, et al. 2014*	RCT	135 PD	(1) Full‐mouth NSPT (2) No treatment	(1) 0.79 ± 0.19 mm^a^ (138 PR) (2) 0.79 ± 0.15 mm^a^ (135 PR)	–	–	^−^	(1) 0.76 ± 0.16 mm^a^ (89 PR) (2) 0.78 ± 0.15 mm^a^ (79 PR)	–
*Kudo C, et al. 2018*	Multicenter single‐arm cohort study	82 PD	NSPT + SPT	0.82 ± 0.23 mm (82 PR)	0.82 ± 0.20 mm (28 PR)	–	–	0.79 ± 0.19 mm (23 PR)	0.94 ± 0.14 mm (8 PR)
*Piconi S, et al. 2009*	Single‐arm cohort study	35 PD	NSPT	0.55 ± 0.03 mm	–	–	0.40 ± 0.04 mm^b^	0.45 ± 0.04 mm^b^	–
*Salah S, et al. 2023*	Single‐arm cohort study	30 PD with rheumatoid arthritis	Full‐mouth NSPT	1.1 ± 0.2 mm	–	–	0.96 ± 0.2 mm^b^	–	–
*Toregeani JF, et al. 2014*	Single‐arm cohort study	21 SPD	NSPT	0.60 ± 0.11 mm	–	–	0.54 ± 0.11 mm^b^	0.58 ± 0.11 mm	–

Abbreviations: CIMT, carotid intima‐media thickness; NSPT, nonsurgical periodontal treatment; PD, periodontitis; RCT, randomized clinical trial; SPD, severe periodontitis; SPT, supportive periodontal treatment; t.i.d., three times a day (*ter in die*).

^a^ Statistically significant difference throughout all follow‐up sessions without distinguishing treatment protocol.

^b^ Statistically significant difference compared to baseline.

### 3.3 Risk of bias results

The results of the Cochrane Rob2 tool for RCT studies have been resumed in Figure S1 in the online *Journal of Periodontology*. One article showed a low risk of bias,^12^ three RCTs presented some concerns,^27^, ^31^, ^32^ and the last three studies had a high risk of bias.^20^, ^28^, ^29^ Regarding Domain 1 (D1) for the randomization process, one study^28^ showed some concerns because baseline patient data were not reported separately for the two groups. Another study^29^ was judged as having a high risk for the randomization process since no one was blinded to the allocation, neither the participants nor the authors. For D2, three articles showed a high risk of bias,^20^, ^28^, ^29^ mainly due to the lack of blinding of participants and operators. For D3, two articles presented a high risk of bias^20^, ^29^ because a substantial number of patients were lost during follow‐up, which could have significantly affected the final results. Regarding D4, two articles^28^, ^29^ were judged as having a high risk of bias because the clinicians were not blinded to the group allocation. For D5, most of the included RCTs^20^, ^27^, ^28^, ^29^, ^31^, ^32^ presented some concerns in the selection of reported results because a prespecified analysis plan finalized before the availability of unblinded outcomes was not described.

**FIGURE 2:**
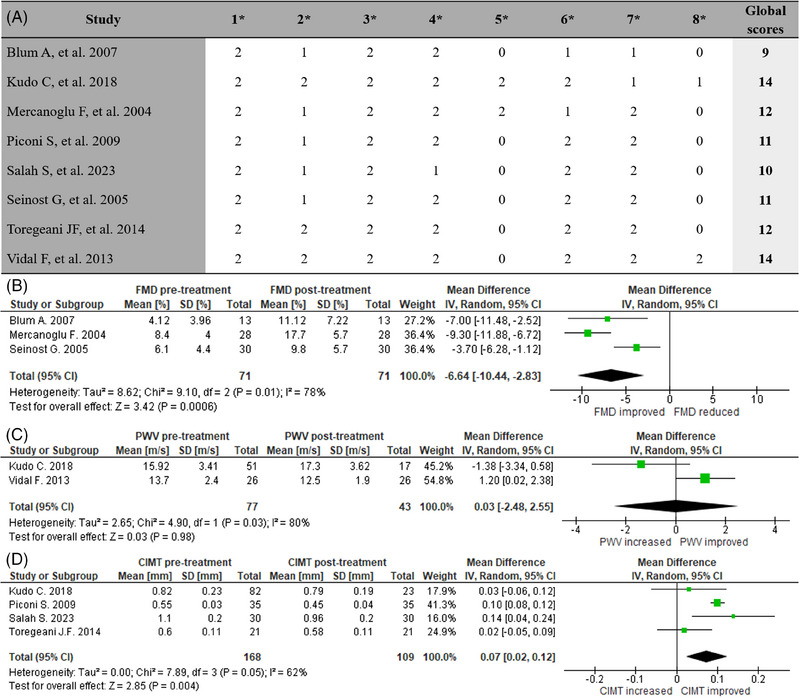
(A) Quality assessment results of single‐arm cohort studies using the MINORS tool composed of eight items (*): (1) a clearly stated aim, (2) inclusion of consecutive patients, (3) prospective collection of data, (4) endpoints appropriate to the aim of the study, (5) unbiased assessment of study endpoint, (6) follow‐up period appropriate to the aim of the study, (7) loss to follow‐up less than 5%, (8) prospective calculation of study size. Each item has been scored as follows: 0 (not reported), 1 (reported but inadequate), and 2 (reported and adequate). The global ideal score for noncomparative studies should be 16. (B) Forest plot for impact of NSPT on FMD (single‐arm cohort studies). (C) Forest plot for impact of NSPT on PWV (single‐arm cohort studies). (D) Forest plot for impact of NSPT on CIMT (single‐arm cohort studies). CIMT, carotid intima‐media thickness; FMD, flow‐mediated dilatation; MINORS, methodological index for nonrandomized studies; NSPT, nonsurgical periodontal treatment; PWV, pulse wave velocity.

The quality assessment results of single‐arm cohort studies using the MINORS tool are presented in Figure 2A. The global scores ranged from 9^26^ to 14.^18^, ^19^ Items 5 and 8 showed the lowest results because the blind evaluation of the main outcomes was not reported and there was no power analysis. Moreover, regarding Item 2, different studies^17^, ^26^, ^30^, ^33^, ^34^ reported only the total number of patients included and the inclusion criteria, but there were no details on how many patients were excluded and the reasons.

### 3.4 Quantitative synthesis

Eight single‐arm cohort studies were statistically comparable for meta‐analysis.^17^, ^18^, ^19^, ^26^, ^30^, ^33^, ^34^, ^35^ In this regard, there was only one RCT^31^ that reported data in terms of mean and standard deviation at baseline and the following sessions without substantial patient loss during follow‐up. The lack of at least two RCTs per parameter with sufficient data prevented their inclusion in the meta‐analysis. Quantitative results of the primary outcomes are presented in Tables 2, 3, 4. A total number of 64 patients with severe PD and 170 with PD were analyzed at baseline and after NSPT.

#### 3.4.1 Meta‐analysis for FMD

Three studies,^26^, ^30^, ^34^ including a total of 71 patients with PD, were finally included in the meta‐analysis for FMD analysis after NSPT (Figure 2B). The pooled MD (95% CI) between pretreatment and post‐treatment FMD values was −6.64% (−10.44%, −2.83%) with a total overall effect statistically significant (*p* = 0.0006) for FMD improvement. However, there was high heterogeneity among studies (*I* ^2^ = 78%).

#### 3.4.2 Meta‐analysis for PWV

Two studies,^18^, ^19^ including a total of 43 patients with PD, were finally included in the meta‐analysis for PWV analysis after NSPT (Figure 2C). The pooled MD (95% CI) between pretreatment and post‐treatment PWV values was 0.03 m/s (−2.48 m/s, 2.55 m/s) with a nonsignificant total overall effect (*p* = 0.98) and high heterogeneity among studies (*I* ^2^ = 80%).

#### 3.4.3 Meta‐analysis for CIMT

Four articles,^17^, ^18^, ^33^, ^35^ including a total of 109 patients with PD, were finally included in the meta‐analysis for CIMT analysis after NSPT (Figure 2D). The pooled MD (95% CI) between pretreatment and post‐treatment CIMT values was 0.07 mm (0.02 mm, 0.12 mm) with a total overall effect statistically significant (*p* = 0.004) for CIMT improvement and moderate heterogeneity among studies (*I* ^2^ = 62%).

## 4 DISCUSSION

The present meta‐analysis was aimed at providing available evidence regarding the effects of NSPT approaches on the improvement of arterial stiffness outcomes related to early endothelial dysfunction in patients with PD. The results showed that NSPT had significant beneficial effects on FMD and CIMT, while the same was not observed for PVW.

However, considering the limitations of the included studies, the high heterogeneity, and the risk of bias, it is impossible to conclude that NSPT effectively improves arterial stiffness.

Several mechanisms have been proposed to explain the relationship between PD and CVDs, including systemic inflammation, molecular mimicry, and direct vascular injury mediated by periodontal pathogens.^36^, ^37^ In this regard, alterations in endothelial function and arterial distensibility were considered valuable predictors of CVDs, including atherosclerosis.^38^ However, taken together, the different pieces of evidence in the current literature converge in reporting a global worsening of outcomes related to endothelial dysfunction (such as PWV, CIMT, and FMD) in patients affected by severe PD.^9^, ^39^, ^40^

Interestingly, some evidence reported FMD as a valuable diagnostic predictor of early CVD and endothelial function. According to a previous meta‐analysis,^41^ the risk of CVD increases by 10% for each unit of FMD reduction. This is consistent with the results of the present study, which were mainly derived from the single‐arm cohort studies and indicate a 6.64% (95% CI, 2.83%–10.44%) mean increase in FMD after periodontal therapy (*p* = 0.0006), albeit with a high heterogeneity among them (*I* ^2^ = 78%). At the same time, it can be noted that the research designs and methods used in the analyzed studies of the present meta‐analysis to evaluate FMD, including follow‐up periods ranging from 6 weeks to 3 months, varied greatly throughout them. Moreover, other clinical trials^31^, ^32^ reported no significant improvement in FMD after NSPT approaches.

Furthermore, the present meta‐analysis showed a mean reduction of 0.07 mm in CIMT after NSPT (*p* = 0.004). The mean estimates of CIMT progression in the general population range from 0.001 to 0.030 mm per year, in agreement with previous evidence.^42^ Research indicates that there is a 1.15‐fold increase in the relative risk of future myocardial infarction and a 1.18‐fold increase in the risk of stroke for every 0.1 mm difference in CIMT.^43^ These findings could indicate a mild protective effect of NSPT from CVD events, but this evidence derives from uncontrolled studies with moderate heterogeneity (*I* ^2^ = 62%), which could have derived from considerable differences in PD diagnosis and CIMT assessment criteria. An RCT^29^ evaluating CIMT after NSPT with a full‐mouth approach reported a mean improvement of 0.003 mm at 1‐year follow‐up; however, the study did not distinguish between interventions and controls in detail and reported results obtained in only half of the initially enrolled patients as several patients were lost to follow‐up.

The results derived from two single‐arm cohort studies showed an overall mean PWV improvement of 0.03 m/s that was not significant (*p* = 0.98) and characterized by high heterogeneity (*I* ^2^ = 80%). In particular, Vidal et al.^19^ observed a noteworthy decrease (1.2 m/s) in PWV values 6 months following NSPT. On the other hand, another trial,^18^ which reported a gradual improvement of PWV during follow‐up, had high rates of loss to follow‐up, while two RCTs^20^, ^28^ did not report nonsignificant variations in PWV at 6‐month and 1‐year sessions, respectively. Moreover, these studies reported mixed PWV results, preventing them from being able to differentiate between interventions and controls. Another RCT,^29^ which distinguished the groups’ results, did not detect significant PWV improvement during follow‐up.

Previous meta‐analyses,^9^, ^16^, ^44^ mainly based on single‐arm cohort studies evaluating the impact of NSPT on PWV, reported that PD patients had increased PWV compared to healthy controls, presenting contradictory results when evaluating the effect of NSPT on PWV through interventional studies. The present meta‐analysis, which aimed to update and further clarify the previous evidence, highlighted that NSPT could not provide beneficial effects on arterial stiffness. Moreover, the most recent clinical studies included^18^, ^20^, ^28^ found a nonsignificant efficacy of NSPT on arterial stiffness outcomes; specifically, even despite beneficial effects on FMD and CIMT, NSPT did not result in improvements in PWV, with a high heterogeneity among the selected and included studies.

A possible biological explanation for the transient impact of NSPT on arterial stiffness outcomes could be the increase in acute transient local and systemic inflammation, with the release of inflammatory mediators following NSPT immediately in the first hours following treatment,^45^ influencing endothelial and arterial functions. However, once the initial acute inflammatory phase elapses, there is a stabilization phase that lasts up to 6 months and is due to the clinical effects of NSPT with the associated reduction in the number of periodontal pockets and associated bleeding on probing (BoP) reduction. This determines a slow but stable decrease in serum inflammatory endothelial biomarkers which, based on the evidence from this study, did not significantly affect arterial stiffness‐related parameter PWV.^12^, ^45^ In this regard, increased CIMT, identified as an early precursor of endothelial dysfunction related to systemic inflammation^46^ together with FMD, has been shown to be influenced by oxidative stress and nitric oxide homeostasis, both of them closely associated with the worsening of PD.^4^, ^6^, ^47^, ^48^ Conversely, PWV, influenced by arterial aging (arteriosclerosis) and blood pressure, may be less sensitive to the systemic fluctuations induced by PD and following NSPT.^49^

In this regard, it appears that NSPT, despite its widely demonstrated medium‐ and long‐term clinical benefits, would seem to lead only to a significant transient alteration of endothelial function, an increase of body temperature, a higher tendency of developing blood clots, and a reduction of renal function, especially in the first stages following NSPT.^45^, ^50^

Furthermore, the high heterogeneity and bias observed across studies underscores the need for caution in interpreting the present results. Heterogeneity may have stemmed from study population variations, periodontal treatment protocols, assessment techniques, and other methodological factors. Indeed, the generalized moderate‐high risk of bias underscores the need for methodologically rigorous research in this field to enhance the reliability and validity of findings. The included evidence reported variability and limitations associated with the arterial stiffness outcome measurement, such as environmental (e.g., the room temperature during measurement and the timing of the exam) and patient‐related features (e.g., patient's blood pressure, consumption of fatty foods or caffeine, comorbidities and infections, or hormonal imbalance),^49^, ^51^ all relevant variables that should be carefully evaluated in the analysis of the results obtained from the various clinical trials, especially when evaluating PWV, a parameter that is considered to be less operator‐dependent and is the gold standard for measuring arterial stiffness but is strictly correlated to the patient's status.

Moreover, RCTs were excluded from the quantitative analysis because they did not meet the final requirements, such as reporting of standard deviation at baseline and at each follow‐up session and the lack of significant loss of patients during the study follow‐up. Thus, evidence from uncontrolled studies alone is considered to be of low quality. Furthermore, the included article with a longer follow‐up period^18^ (up to 3 years) presented high rates of loss to follow‐up, preventing the achievement of reliable long‐term results.

## 5 CONCLUSION

The present study provides moderate evidence for the potential beneficial effects of NSPT on FMD and CIMT, but not on PWV. However, the limitations in the study design, including the lack of control groups, the high risk of bias, and overall heterogeneity, tempered the results' strength. In this regard, further clinical trials, possibly with an RCT design, homogeneous inclusion, and rigorous criteria are necessary to achieve more robust conclusions and fully understand the implications of periodontal treatment in endothelial functions and CVDs in patients with PD.

## AUTHOR CONTRIBUTIONS

Alessandro Polizzi and Gaetano Isola conceived the research, performed the article screening, and wrote the manuscript. Luigi Nibali and Gianluca Martino Tartaglia performed the article screening, revised the manuscript, and critically revised the data.

## CONFLICT OF INTEREST STATEMENT

The authors declare that they have no conflict of interest in relation to the present study.

## Supporting information



Supporting Information

Supporting Information

## Data Availability

The data that support the findings of this study are available on reasonable request from the corresponding author.

## Footnotes

* Review Manager 5.4; Cochrane Collaboration, London, UK.
