# Supplementary figures and images for: Impact of nonsurgical periodontal treatment on arterial stiffness outcomes related to endothelial dysfunction: A systematic review and meta‐analysis
Source: J Periodontol. 2024 Nov 16;96(4):330–45. doi: 10.1002/JPER.24-0422 (PMC12062727; doi:10.1002/JPER.24-0422)

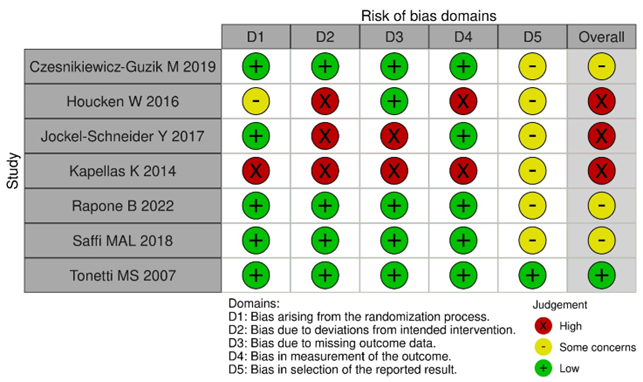

Supplement: Supplementary file 1 — Supporting Information [file JPER-96-330-s002.tif]
