# Supplementary material for: Impact of nonsurgical periodontal treatment on arterial stiffness outcomes related to endothelial dysfunction: A systematic review and meta‐analysis
Source: J Periodontol. 2024 Nov 16;96(4):330–45. doi: 10.1002/JPER.24-0422 (PMC12062727; doi:10.1002/JPER.24-0422)
Supplement: Supplementary file 2 — Supporting Information [file JPER-96-330-s001.docx]

**Supplementary Table 2**. Searching strategy in the different databases.

| **Database** | **Boolean Operator** | **Results** |
| --- | --- | --- |
| Pubmed | ((Periodontitis [Mesh Terms]) OR "periodontal therapy" OR "non-surgical periodontal treatment" OR "scaling and root planing" OR "scaling" OR "debridement") AND ("endothelial dysfunction" OR ("arterial stiffness" [Mesh Terms]) OR "pulse wave velocity" OR "flow-mediated dilatation" OR "flow-mediated dilation" OR "carotid intima-media thickness") | 178 |
| Scopus | (Periodontitis OR "periodontal therapy" OR "non-surgical periodontal treatment" OR "scaling and root planing" OR "scaling" OR "debridement") AND ("endothelial dysfunction" OR "arterial stiffness" OR "pulse wave velocity" OR "flow-mediated dilatation" OR "flow-mediated dilation" OR "carotid intima-media thickness") | 355 |
| Web of Science | (Periodontitis OR "periodontal therapy" OR "non-surgical periodontal treatment" OR "scaling and root planing" OR "scaling" OR "debridement") AND ("endothelial dysfunction" OR "arterial stiffness" OR "pulse wave velocity" OR "flow-mediated dilatation" OR "flow-mediated dilation" OR "carotid intima-media thickness") | 359 |
| LILACS | (Periodontitis OR "periodontal disease" OR "periodontal therapy" OR "non-surgical periodontal treatment" OR "scaling" OR "root planing" OR “debridement”) AND ("endothelial dysfunction" OR "arterial stiffness" OR "pulse wave velocity" OR "flow-mediated dilatation" OR "flow-mediated dilation" OR "carotid intima-media thickness") | 22 |
| **Total** |  | **914** |

**Supplementary Table 3**. Articles excluded after full-text evaluation.

| **Article** | **Reason for exclusion** |
| --- | --- |
| Kapellas, K., et al. "The Effect of Periodontal Therapy on Carotid Intima-Media Thickness among Aboriginal Australians: A Randomised Controlled Trial." International Journal of Epidemiology 44.suppl_1 (2015): i140-i141. | Conference paper |
| Carallo, C., De Franceschi, M. S., Tripolino, C., Iovane, C., Catalano, S., Giudice, A., ... & Gnasso, A. (2015). Periodontal treatment elevates carotid wall shear stress in the medium term. Medicine, 94(42). | Main outcomes not included |
| Li, X., Tse, H. F., Yiu, K. H., Li, L. S. W., & Jin, L. (2011). Effect of periodontal treatment on circulating CD34+ cells and peripheral vascular endothelial function: a randomized controlled trial. Journal of clinical periodontology, 38(2), 148-156. | Main outcomes not included |
| Ramírez, J. H., Arce, R. M., & Contreras, A. (2011). Periodontal treatment effects on endothelial function and cardiovascular disease biomarkers in subjects with chronic periodontitis: protocol for a randomized clinical trial. Trials, 12(1), 1-10. | Study protocol |
| Seinost, G., Horina, A., Arefnia, B., Kulnik, R., Kerschbaumer, S., Quehenberger, F., ... & Wimmer, G. (2020). Periodontal treatment and vascular inflammation in patients with advanced peripheral arterial disease: a randomized controlled trial. Atherosclerosis, 313, 60-69. | Main outcomes not included |
| Skilton, M. R., Maple-Brown, L. J., Kapellas, K., Celermajer, D. S., Bartold, M., Brown, A., ... & Jamieson, L. M. (2011). The effect of a periodontal intervention on cardiovascular risk markers in Indigenous Australians with periodontal disease: the PerioCardio study. BMC public health, 11(1), 1-8. | Study protocol |

**Supplementary Table 4.** Quality assessment results of single-arm cohort studies using the MINORS tool composed by 8 items*: (1) a clearly stated aim, (2) inclusion of consecutive patients, (3) prospective collection of data, (4) endpoints appropriate to the aim of the study, (5) unbiased assessment of the study endpoint, (6) follow-up period appropriate to the aim of the study, (7) loss to follow-up less than 5%, (8) prospective calculation of the study size. Each item has been scored as follows: 0 (not reported), 1 (reported but inadequate) and 2 (reported and adequate). The global ideal score for non-comparative studies should be 16.

| **Study** | **1*** | **2*** | **3*** | **4*** | **5*** | **6*** | **7*** | **8*** | **Global scores** |
| --- | --- | --- | --- | --- | --- | --- | --- | --- | --- |
| Blum A, et al. 2007 ^27^ | 2 | 1 | 2 | 2 | 0 | 1 | 1 | 0 | 9 |
| Kudo C, et al. 2018 ^32^ | 2 | 2 | 2 | 2 | 2 | 2 | 1 | 1 | 14 |
| Mercanoglu F, et al. 2004 ^33^ | 2 | 1 | 2 | 2 | 2 | 1 | 2 | 0 | 12 |
| Piconi S, et al. 2009 ^34^ | 2 | 1 | 2 | 2 | 0 | 2 | 2 | 0 | 11 |
| Salah S, et al. 2023 ^37^ | 2 | 1 | 2 | 1 | 0 | 2 | 2 | 0 | 10 |
| Seinost G, et al. 2005 ^38^ | 2 | 1 | 2 | 2 | 0 | 2 | 2 | 0 | 11 |
| Toregeani JF, et al. 2014 ^40^ | 2 | 2 | 2 | 2 | 0 | 2 | 2 | 0 | 12 |
| Vidal F, et al. 2013 ^41^ | 2 | 2 | 2 | 2 | 0 | 2 | 2 | 2 | 14 |
